# Electronic applications for the CFQ-R scoring

**DOI:** 10.1186/s12931-017-0592-z

**Published:** 2017-05-30

**Authors:** Andreas Ronit, Marco Gelpi, Jonathan Argentiero, Inger Mathiesen, Susanne D. Nielsen, Tanja Pressler, Alexandra L. Quittner

**Affiliations:** 1 Department of Infectious Diseases 8632, University Hospital of Copenhagen, Copenhagen, Denmark; 2 Copenhagen Cystic Fibrosis Center, Department of Infectious Diseases, University Hospital of Copenhagen, Copenhagen, Denmark; 3 Behaviroal Health Systems Research, Miami, FL USA

**Keywords:** Application, Cystic fibrosis, Patient reported outcomes, Software

## Abstract

Patient reported outcomes (PROs) have become widely accepted outcome measures in cystic fibrosis (CF) and other respiratory diseases. The Cystic Fibrosis-Questionnaire-Revised (CFQ-R) is the best validated and most widely used PRO for CF. Data collection can be time-intensive, and electronic platforms would greatly facilitate the feasibility, utility and accuracy of administration of the CFQ-R. Given that the CFQ-R is utilized in virtually all clinical trials worldwide and is increasingly integrated into clinical practice, we developed a software application that will help users to administer, score and save CFQ-R data for all versions. All codes are open access, which will enable other PRO users to design similar applications for other respiratory diseases, such as primary ciliary dyskinesia and non-CF bronchiectasis.

## Background

Patient-reported outcomes (PROs), are defined as systematic assessments of symptoms or daily functioning as reported by the patient, him or herself [1]. They are important endpoints in clinical trials of new medications and can serve as outcome measures in clinical research and to improve clinical care. Both the U.S. Food and Drug Administration (FDA) and European Medicines Agency (EMA) have formally recognized their importance [1, 2] and thus, PROs are increasingly utilized as primary or secondary outcomes in the drug approval process [3]. Numerous studies have also demonstrated the importance of integrating them into clinical practice and research to monitor the health of a population, evaluate quality of care, and aid in communication and clinical decision making [4, 5]. Historically, the mode of administration for PROs has been by hard copy/paper format. This can be a time- and resource intensive process in a clinic setting and therefore, our main objective was to develop an electronic version to reduce the time associated with data entry and scoring, and reduce errors both in the administration (e.g., skipping items) and data entry processes.

## Main text

The use of PROs has increased significantly in cystic fibrosis (CF) research and care over the past 20 years [6]. The Cystic Fibrosis Questionnaire-Revised (CFQ-R) is the most commonly used and best validated disease-specific PRO [7–9]. The CFQ-R has been validated (i.e. demonstrated reliability, validity, and sensitivity) and has been translated into 38 languages [8], and has been used in clinical trials evaluating medications with differing mechanisms of action (e.g., hypertonic saline, inhaled antibiotics, CFTR correctors-modulators). Importantly, it was developed using the FDA Guidance (2009), including qualitative phases, which elicited patient, parent and provider input. It was designed to systematically measure respiratory and gastrointestinal symptoms, treatment burden, and daily functioning.

Four validated versions of the CFQ-R were adapted for electronic administration in this study: 1) self-report for adolescents and adults, ages 14 years and older (CFQ-R Teen/Adult); 2) self-report for children, ages 12–13; 3) interviewer-administered version for children ages 6 to 11; and 4) parent proxy version for caregivers of children, ages 6 to 13 years.

The code of the application is open source and can be found at https://github.com/CFQR where continuous integration is achieved with Travis CI. The CFQ-R-application is a React web application (HTML, CSS, Javascript) built with ReactJS framework. The CFQ-R application is freely available at https://cfqr.github.io and is made to be run from different environments: It can be used online, downloaded as a desktop application for offline use (Windows, Mac, Linux), and is also available as a mobile application for iOS and Android platforms (from popular application stores).

**Fig. 1:**
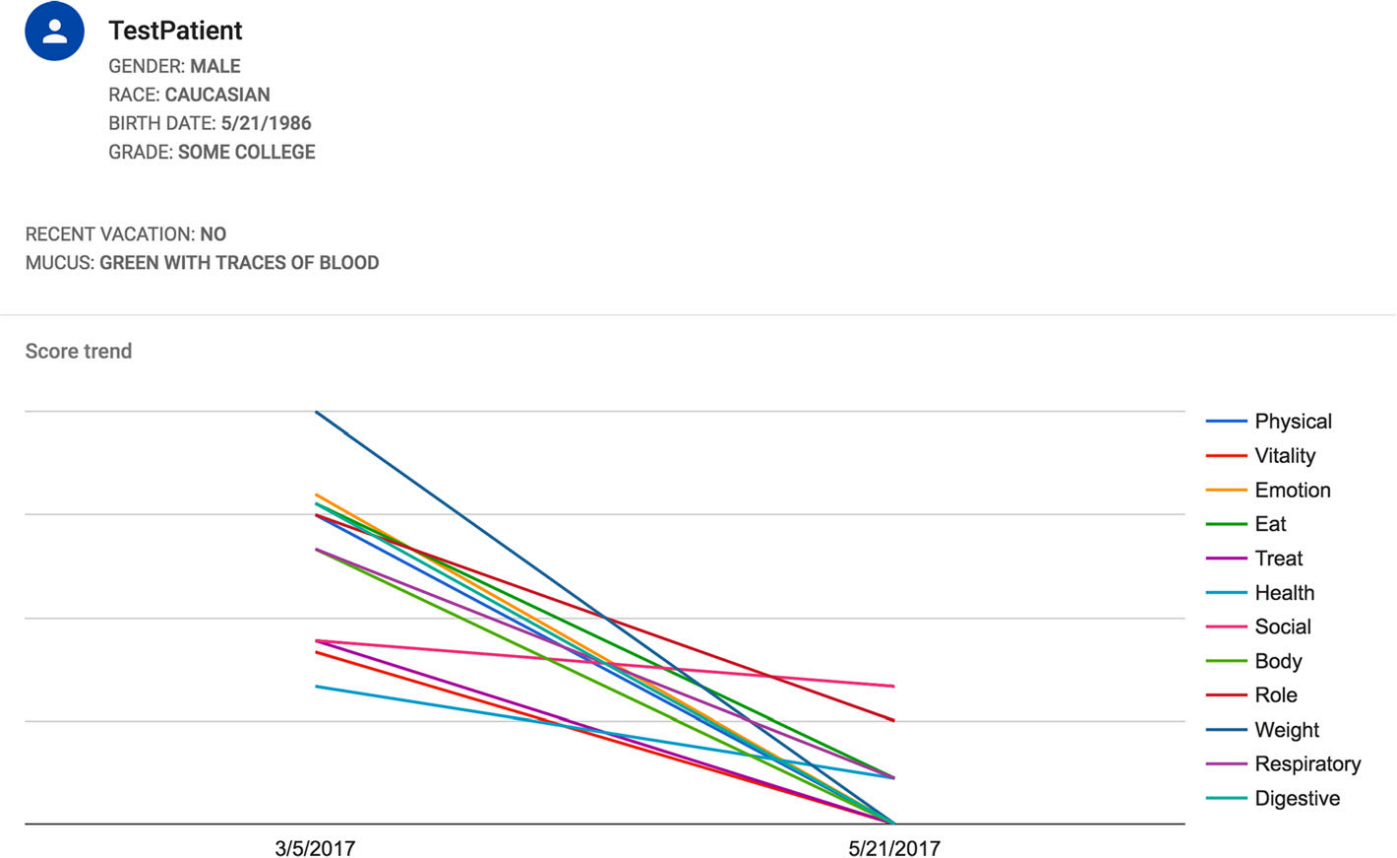
Cystic fibrosis questionnaire revised (CFQ-R) application output. Output from the CFQ-R application (teen/adult version) showing demographic data and trajectories for 12 domains. The application calculates the score derived from the CFQ-R, on a 0–100 scale with higher scores indicating better health status. Other output forms are also available

Besides automatically calculating the scores from the different domains obtained by the CFQ-R, saving time, and avoiding miscalculations, the CFQ-R application has several other functionalities. It can be used to visually represent longitudinal trajectories of CFQ-R scores (Fig. 1), which may be helpful for the clinician. It also allows data export for downstream statistical processing for the researcher. The application can store data in different ways. Data can be stored locally on the tablet or PC used by the user. The user may also wish to connect the device to the Internet to allow automatic backup in a cloud (using the Google service Firebase). The latter function will enable synchronized data capture from multiple devices (e.g. in larger clinical trial). CFQ-R’s in eight languages have currently been included (i.e. Danish, Dutch, English, German, Italian, French, Spanish, and Swedish). Other languages can be added on request.

As an additional perspective, users of non-CF PROs measures may wish to use codes from this application as a template to make similar tools that can be used for other respiratory diseases, such as primary ciliary dyskinesia and non-CF bronchiectasis. Although a simpler desktop tool for the St. George’s Respiratory Questionnaire was previously developed [10], we are not aware of the existence of similar applications for other respiratory PROs.

This application has some limitations. First, the user should be aware that data collection has to comply with the rules established by local ethics committees. Second, although a meta-analysis of PROs has concluded that there is no bias attributable to paper vs. electronic completion of a large number of PROs [11], we have not studied the user experience for this application of the CFQ-R.

## Conclusion

In summary, utilization and integration of PROs into both research and clinical practice is facilitated by the availability of electronic modes of administration. Thus, this electronic application was primarily developed to make both in-clinic use of CFQ-R easier and more efficient, and to facilitate accurate and large-scale data collection with the CFQ-R. An electronic platform enables the clinician to immediately score the CFQ-R and utilize the scores to discuss concerns with patients. Use of an electronic version of the CFQ-R will also enable the generation of high-quality data, e.g. larger amounts of data, consistent data collection, and high response rates. The CFQ-R is used in virtually every clinical trial of new medications and treatments in CF and the electronic platform facilitates this type of data collection across countries and languages.

## Abbreviations

CFQ-R: Cystic fibrosis questionnaire revised
EMA: European Medicines Agency
FDA: Food and drug administration
PRO: Patient reported outcomes
